# Annotation-Free Deep Learning-Based Prediction of Thyroid Molecular Cancer Biomarker BRAF (V600E) from Cytological Slides

**DOI:** 10.3390/ijms24032521

**Published:** 2023-01-28

**Authors:** Ching-Wei Wang, Hikam Muzakky, Yu-Ching Lee, Yi-Jia Lin, Tai-Kuang Chao

**Affiliations:** 1 Graduate Institute of Biomedical Engineering, National Taiwan University of Science and Technology, Taipei 106335, Taiwan; 2 Graduate Institute of Applied Science and Technology, National Taiwan University of Science and Technology, Taipei 106335, Taiwan; 3 Department of Pathology, Tri-Service General Hospital, Taipei 106335, Taiwan; 4 Institute of Pathology and Parasitology, National Defense Medical Center, Taipei 106335, Taiwan

**Keywords:** papillary thyroid cancer, fine needle aspiration cytology, BRAF (V600E) prediction, deep learning, weakly supervised, multiple instance learning, CLAM, precision oncology

## Abstract

Thyroid cancer is the most common endocrine cancer. Papillary thyroid cancer (PTC) is the most prevalent form of malignancy among all thyroid cancers arising from follicular cells. Fine needle aspiration cytology (FNAC) is a non-invasive method regarded as the most cost-effective and accurate diagnostic method of choice in diagnosing PTC. Identification of BRAF (V600E) mutation in thyroid neoplasia may be beneficial because it is specific for malignancy, implies a worse prognosis, and is the target for selective BRAF inhibitors. To the authors’ best knowledge, this is the first automated precision oncology framework effectively predict BRAF (V600E) immunostaining result in thyroidectomy specimen directly from Papanicolaou-stained thyroid fine-needle aspiration cytology and ThinPrep cytological slides, which is helpful for novel targeted therapies and prognosis prediction. The proposed deep learning (DL) framework is evaluated on a dataset of 118 whole slide images. The results show that the proposed DL-based technique achieves an accuracy of 87%, a precision of 94%, a sensitivity of 91%, a specificity of 71% and a mean of sensitivity and specificity at 81% and outperformed three state-of-the-art deep learning approaches. This study demonstrates the feasibility of DL-based prediction of critical molecular features in cytological slides, which not only aid in accurate diagnosis but also provide useful information in guiding clinical decision-making in patients with thyroid cancer. With the accumulation of data and the continuous advancement of technology, the performance of DL systems is expected to be improved in the near future. Therefore, we expect that DL can provide a cost-effective and time-effective alternative tool for patients in the era of precision oncology.

## 1. Introduction

Papillary thyroid cancer (PTC) is the most prevalent form of malignancy among all thyroid cancers. PTC is usually contained within the thyroid gland and is generally biologically indolent. 80% of all cases are cured after radical surgery and radioiodine ablative treatment [1]. However, more than 25% of patients with PTC developed a recurrence during a long-term follow-up [2]. Approximately 5% of PTC are diagnosed with radioactive iodine-refractory (RAI-R) disease, which is associated with a significantly poorer outcome [3]. Chemotherapy plays no significant role in the systemic treatment of advanced differentiated thyroid cancer [1].

The discovery of molecular biomarkers for thyroid cancer has significantly improved the understanding of thyroid cancer pathogenesis, leading to more personalized treatment for thyroid cancer patients [4]. Studies of molecular genetic alterations provide better guidance for understanding PTC progression and therapeutic directions. Medical treatment is currently based on tyrosine kinase inhibitors as targeted molecular therapies, based on studies showing that the dedifferentiation of cells resulting in unresponsiveness to RAI therapy correlates with the degree of mitogen-activated protein kinase (MAPK) activation [5]. BRAF is the only member of the RAF family activated by mutation in human cancers. PTC is often characterized by BRAF mutations (mainly V600E) with more aggressive and iodine-resistant phenotypes, which trigger activation of the MAPK cascade [2,6,7]. Vemurafenib and dabrafenib, two selective BRAF inhibitors that restore RAI uptake and efficacy, demonstrated a partial response in many metastatic or unresectable radioiodine-resistant BRAF(V600E)-mutated PTC [8,9,10].

Immunostaining, a long-used and indispensable tool, is a routine technique used in surgical pathology. It provides an easy, cheap, and widely available technique for differential diagnosis and molecular testing to detect genetic alterations at the protein level [11]. Immunostaining for thyroid cancer BRAF (V600E) has shown excellent concordance with molecular testing [12,13,14]. Liquid biopsy is a non-invasive method used for early diagnosis, follow-up and molecular profiling of cancer [15]. Fine-needle aspiration cytology (FNAC) is regarded as the most important diagnostic tool for thyroid lesions because of its simplicity, safety, and cost-effectiveness [16]. Liquid-based ThinPrep (TP) cytology uses a filtration process and thin-layer cell deposition. This semiautomated device has recently been widely used to diagnose PTC with high diagnostic sensitivity and excellent cell preservation [17,18,19].

The Deep learning (DL)-based algorithms have been developed for various tasks involved in tumor pathology, including tumor diagnosis, subtyping, grading, staging, and prognosis prediction, as well as the identification of pathological features, biomarkers, and genetic change [20]. Machine learning techniques also promise better drug response predictions [21]. Here we have built DL-based approaches to accurately predict BRAF (V600E) results in cytological slides. The experimental finding suggests that the DL model may be helpful for personalized medicine and may effectively predict BRAF (V600E). Eventually, the DL framework prediction results directly from Papanicolaou-stained thyroid FNAC and TP cytological slides, which are helpful for novel BRAF inhibitor targeted therapies and prognosis prediction.

## 2. Results

### 2.1. Evaluation Metrics

For quantitative evaluation, we utilize the accuracy, precision, sensitivity, specificity, and mean SS to compare and assess the performance of the benchmark approaches and the modified method. The metrics are calculated as follows:

(1) $$\;\begin{array}{c} \mathrm{Accuracy}=\frac{\mathrm{TP}+\mathrm{TN}}{\mathrm{TP}+\mathrm{TN}+\mathrm{FP}+\mathrm{FN}} \end{array}$$

(2) $$\;\begin{array}{c} \mathrm{Precision}=\frac{\mathrm{TP}}{\mathrm{TP}+\mathrm{FP}} \end{array}$$

(3) $$\;\begin{array}{c} \mathrm{Sensitivity}=\frac{\mathrm{TP}}{\mathrm{TP}+\mathrm{FN}} \end{array}$$

(4) $$\;\begin{array}{c} \mathrm{Specificity}=\frac{\mathrm{TN}}{\mathrm{TN}+\mathrm{FP}} \end{array}$$

(5) $$\;\begin{array}{c} \mathrm{Mean}\;\mathrm{SS}\;(\mathrm{sensitivity},\mathrm{specificity})=\frac{\left(\frac{\mathrm{TP}}{\mathrm{TP}+\mathrm{FN}}\right)+\left(\frac{\mathrm{TN}}{\mathrm{TN}+\mathrm{FP}}\right)}{2} \end{array}$$

where TP denotes the true positive, TN represents the true negative, FP is false positive, and FN indicates the false negative.

### 2.2. Quantitave Analysis

The quantitative evaluation results in classification of BRAF (V600E) status of the individual patient using thyroid FNA and TP slides are presented in Table 1. Overall, the proposed CLAM model is demonstrated to achieve a decent performance, obtaining 87% for accuracy, 94% precision, 91% sensitivity, 71% specificity, and 81% mean SS, respectively. On the other hand, three benchmark models obtained high sensitivity greater than 90% but comparably low specificity at 0%, 14%, and 29%, respectively, which might be caused by class imbalance in the data set where there are 82% positive samples with 18% negative samples. In comparison, the proposed model is demonstrated to tackle this limitation by acquiring the highest specificity at 71%. Results from the quantitative evaluation show that the modified model outperformed the three state-of-the-art benchmark methods, including NASNetLarge [22], MIL with Resnet34 + RNN [23], and the original CLAM [24].

## 3. Materials and Methods

### 3.1. The Datasets

De-identified and digitized 118 WSIs, including 107 PTC cytologic slides (smear, Papanicolaou stained, *n* = 107) and 11 PTC cytologic slides (ThinPrep, Papanicolaou stained, *n* = 11) were collected from the Department of Pathology, Tri-Service General Hospital, National Defense Medical Center, Taipei, Taiwan. All PTC were cytologically diagnosed, accompanied by cytologically confirmed by the two expert pathologists. All patients underwent thyroidectomy within three months to confirm the presence of PTC, while immunohistochemistry (IHC) recorded positive or negative results for BRAF (V600E). Papanicolaou-stained and TP cytological slides of patients with PTC were collected for DL analysis to predict BRAF (V600E) results. Ethical approvals have been obtained from the research ethics committee of the Tri-Service General Hospital (TSGHIRB No.1-107-05-171 and No.B202005070), and the data were de-identified and used for a retrospective study without impacting patient care. All the stained slides were scanned using Leica AT Turbo (Leica, Germany) at 200× overall magnification (with a 20× objective lens). The average slide dimensions are 77,338 × 37,285 pixels with physical size 51.13 × 23.21 mm${}^{2}$. The training model utilizes 79 Papanicolaou-stained WSIs (67%), and the remaining 39 Papanicolaou-stained WSIs (33%) are used as an independent testing set for evaluation.

### 3.2. Methods

We examined three recently state-of-the-art DL models and construct a clustering-constrained-attention multiple-instance learning (CLAM)-based model for classification BRAF (V600E) status of the individual patient using cytological slides. The benchmark models include NASNetLarge [22], multiple instance learning (MIL) with Resnet34 + recurrent neural network (RNN) [23], and CLAM with Resnet50 [24], which have been demonstrated to be successful in computational pathology. In 2020, Tolkach et al. [22] introduced a NasNetLarge-based model for Gleason pattern (GP) classification in prostate cancer patients with an overall accuracy of more than 98%. This research utilized detailed pixel-wise annotations by three expert pathologists to identify the tumor patches, and then the patch-based annotations that form from GP WSIs are utilized. Next, the patch-wise annotations are used to annotate regions within WSIs where the primary Gleason pattern is distinctive from the secondary Gleason pattern. Apart from slide labels, NasNetLarge requires detailed image annotations for fully supervised learning.

In 2019, a weakly-supervised model, i.e., MIL with Resnet34 + RNN, was presented for classification of prostate cancer, basal cell carcinoma, and breast cancer metastases by Campanella et al. [23], and the main strength of this approach is that it requires slide labels only without annotating WSIs at the image level. This weakly-supervised model resulted in areas under the curve above 0.98 for all cancer types to clinically validate the generalization model performance. The MIL strategy trains a deep learning network with rich tile-wise feature representations, aggregates the information across WSIs and makes a final diagnosis by RNN pooling-based mechanisms.

In 2021, Lu et al. [24] proposed an improved MIL-based technique, CLAM, that regards each slide as a collection of many patches or instances. As the original MIL with Resnet34 + RNN [23] shows limited or no improvement using RNN-based aggregation on three various large datasets (prostate cancer, skin cancer basal cell carcinoma, and lymph node metastasis detection), instead of utilizing RNN as an aggregator, CLAM adopts an attention-based pooling MIL formulation to tackle the stagnation of AUC limitation. DL models trained using CLAM are demonstrated effective for independent data sources, biopsy slides, various scanning modalities, and smartphone microscopy images without domain adaptation or fine-tuning [24]. These crucial properties abovementioned are the main reason that CLAM is adopted as the backbone method to construct the improved version for this study.

### 3.3. Proposed CLAM-Based Method

Conventional convolutional neural networks (CNNs) consist of several levels of convolution nodes, pooling layers, and fully connected layers. We utilized Resnet101 [25] as a feature extractor on our modified CLAM-based network to transform the foreground patches into sets of low-dimensional feature representation. This architecture applies residual blocks made of shortcut connections that perform identity mapping and add their outputs to the outputs of the stacked layers. Resnet101 comprises one max pooling layer followed by 48 stacks of residual blocks (99 convolutional layers), then ends with a fully connected layer and a softmax output layer.

**Figure 1:**
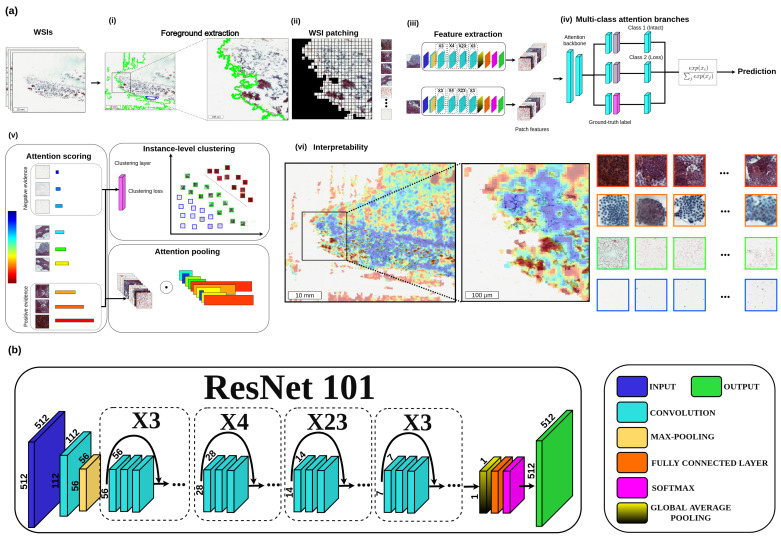
The proposed annotation-free deep learning framework for classification BRAF (V600E) status from Papanicolaou-stained thyroid FNA and TP WSIs. (**a**) (**i**) Segmentation to get the foreground. (**ii**) WSI patching process. (**iii**) Feature extraction process. (**iv**) Aggregating patch-based information into slide-level representations. (**v**) The attention network ranks each region in the slide and assigns an attention score based on its relative importance to the slide-level diagnosis (left). Attention pooling weighs patches by their respective attention scores and summarizes patch-level features into slide-level representations (bottom right). Strongly patched (red) and weakly patched (blue) regions as representative samples to supervise clustering layers in separating between the positive and negative instances of distinct classes (top right). (**vi**) The attention scores can be visualized as a heatmap to identify ROIs and interpret the vital morphology used for diagnosis. (**b**) The detailed architecture of the proposed deep learning model.

Figure 1a shows the workflow of the proposed framework. Initially, (i) each WSI is segmented into the foreground region of each slide, and (ii) divides each slide into many smaller patches (for example, 256 × 256 pixels). (iii) Through feature extraction, all foreground patches are converted into sets of low-dimensional feature embeddings to be fed to the attention network. (iv) Then, the attention network aggregates patch-wise evidence into slide-level representations, which are then used to create the diagnostic prediction. (v) After the slide-wise representations are obtained, the attention network ranks each region in the slide, and an attention score is formed based on its relative importance to the slide-wise diagnosis. Next, Attention pooling weighs patches by their respective attention scores and summarizes patch-level features into slide-level representations. Consequently, strongly patched (denoted by red regions) and weakly patched (represented by blue regions) are representative samples to supervise the clustering process that separates positive and negative instances. (vi) Heatmap visualization can be formed from the attention scores to identify ROIs and interpret the vital morphology used for diagnosis. Figure 1b presents the detailed architecture of the proposed deep learning model as a feature extractor.

Initially, the first fully connected layer $W_{1}\in R^{512\times 1024}$ further squeezes each fixed patch-level representation $z_{k}\in R^{1024}$ to a 512-dimensional vector $h_{k}=W_{1}z_{k}$. The attention network consists of several stacked fully connected layers; if we consider the first two layers of the attention network $U_{a}\in R^{256\times 512}$ and $V_{a}\in R^{256\times 512}$ and $W_{1}$ collectively as part of the attention backbone shared by all classes, the attention network then splits into *N* parallel attention branches $W_{a,1},\cdots ,W_{a,N}\in R^{1x256}$. Correspondingly, *N* parallel independent classifers $W_{c,1},\cdots ,W_{c,N}$ are constructed to score each class-specifc slide-level representation. Consequently, the attention score of the *k*th patch for the *i*th class, denoted $a_{i,k}$, is given by Equation (6):

(6) $$\;\begin{array}{c} \frac{\exp\{W_{a,i}\left(\tanh\left(V_{a}h_{k}\right)⊙\mathrm{sigm}\left(U_{a}h_{k}\right)\right)\}}{\sum_{j=1}^{K}\exp\left\{W_{a,i}\left(\tanh\left(V_{a}h_{j}\right)⊙\mathrm{sigm}\left(U_{a}h_{j}\right)\right)\right\}} \end{array}$$

The slide-level representation aggregated per the attention score distribution for the *i*th class, denoted $h_{\mathrm{slide},i}\in R^{512}$, is given by Equation (2):

(7) $$\;\begin{array}{c} h_{\mathrm{slide},i}=\sum_{k=1}^{K}a_{i,k}h_{k} \end{array}$$

The corresponding unnormalized slide-level score $s_{\mathrm{slide},i}$ is given via the classifier layer $W_{c,1}\in R^{1x256}$ by $s_{\mathrm{slide},i}=W_{c,i}h_{\mathrm{slide},i}$. We utilized dropout (*p* = 0.25) after each layer in the attention backbone of the model for regularization. For inference the predicted probability distribution over each class is computed by applying a softmax function to the slide-level prediction scores $s_{\mathrm{slide}}$

To further encourage the learning of class-specific features, we introduce an additional binary clustering objective during training. For each of *N* classes, we put a fully connected layer after the first layer $W_{1}$. If we denote the weight of the clustering layer that corresponds to the ith class as $W_{\mathrm{inst},1}\in R^{2x512}$, the cluster assignment scores predicted for the *k*th patch, showed by $p_{i,k}$ is given as:

(8) $$\;\begin{array}{c} p_{i,k}=W_{\mathrm{inst},i}h_{k} \end{array}$$

For the instance-level clustering task, *N*-class classification problem, neural network models output a vector of prediction scores **s**, where each entry in **s** corresponds to the prediction of the model for a single class made. Given the set of all possible ground-truth labels Y={1,2,3,⋯,N} and ground-truth label y∈Y, the multi-class support vector machine (SVM) loss penalizes the classifier linearly in the difference between the prediction score for the ground-truth class and the highest prediction score for the remaining classes only if that difference is greater than a specified margin α (Equation (9)). The smoothed variant (Equation (10)) adds a temperature scaling τ to the multi-class SVM loss, with which it has been shown to be infinitely differentiable with non-sparse gradients and suitable for the optimization of deep neural networks when the algorithm is implemented efficiently. The smooth SVM loss can be considered as a generalization of the widely used cross-entropy classification loss for different choices of finite values for the margin and different temperature scaling

(9) $$\;\begin{array}{c} l\left(s,y\right)=max\{\max_{j\in Y\backslash \{y\}}\left\{s_{j}+\alpha \right\}-s_{y},0\} \end{array}$$

(10) $$\;\begin{array}{c} L_{1,\tau }\left(s,y\right)=\tau \log\left[\sum_{j\in Y}\exp\left(\frac{1}{\tau }\left(\alpha 𝟙\left(j\neq y\right)+s_{j}-s_{y}\right)\right)\right] \end{array}$$

The total loss for a given slide $L_{\mathrm{total}}$ is the sum of both the slide-level classification loss $L_{\mathrm{slide}}$ and the instance-level clustering loss $L_{\mathrm{patch}}$ with optional scaling via scalar $c_{1}$ and $c_{2}$:

(11) $$\;\begin{array}{c} L_{\mathrm{total}}=c_{1}L_{\mathrm{slide}}+c_{2}L_{\mathrm{patch}} \end{array}$$

To compute $L_{\mathrm{slide}}$, $s_{\mathrm{slide}}$ is compared with the ground-truth slide-level label using the standard cross-entropy loss, and to compute $L_{\mathrm{patch}}$, the instance-level clustering prediction scores $p_{k}$ for each sampled patch are compared against their corresponding pseudo-cluster labels using the binary smooth SVM loss.

### 3.4. Implementation Details

The tailored method employs the Resnet101 model as the backbone for training in CLAM-based method. During training, the patches are randomly sampled from slides in the training set using a batch size of 512. We utilize an early stopping mechanism on the model when the validation loss does not drop for 20 consecutive validation epochs, and use the cross-entropy loss function. The model checkpoint with the smallest validation loss is selected for evaluation on the test set, which is consistent with the model selection criteria. Next, the model parameters are optimized via stochastic gradient descent (SGD) using the Adam optimizer with a learning rate of $2\times 10^{-4}$ and weight decay of $1\times 10^{-5}$, with $\beta_{1}$ of 0.9, $\beta_{2}$ of 0.999, E value of $1\times 10^{-8}$, and a dropout ratio of 0.25. The benchmark methods i.e., NASNetLarge [22], MIL with Resnet34 + RNN [23], and CLAM [24] are tested with the original method implementation (https://github.com/gagarin37/deep_learning_pca) accessed on 11 May 2022, (https://github.com/MSKCC-Computational-Pathology/MIL-nature-medicine-2019) accessed on 17 August 2022, and (https://github.com/mahmoodlab/CLAM) accessed on 25 October 2022, respectively.

## 4. Discussion and Conclusions

Thyroid carcinomas can be divided into two broad categories based on their origin follicular epithelial cells or parafollicular C cells. Follicular cell-derived carcinomas are classified into four subtypes, including PTC, follicular carcinoma, poorly differentiated carcinoma, and anaplastic carcinoma [26]. Surgical resection is the standard treatment for most patients with thyroid cancer [27]. PTC can be reliably diagnosed by cytological examination, such as papillary-like features, intranuclear pseudoinclusions, nuclear grooving, and fine, pale chromatin [16].

The BRAF gene is located on chromosome 7q23 and encodes a 95 kDa protein that belongs to the RAF family of tryptophan/serine kinases. T to A mutation at 1799 of the BRAF gene can cause glutamic acid to valine (V600E) point mutation in the encoded protein, activating MAPK pathway, playing a central role in the carcinogenesis of thyroid carcinoma [28,29]. The essential proteins in this pathway are receptor tyrosine kinases. The cascade of downstream events in this pathway ultimately leads to altered cell proliferation, differentiation, and survival, leading to various forms of thyroid carcinoma [26,30,31]. Identification of BRAF (V600E) is important for targeted therapy [32]. The gold standard for determination of BRAF status is tissue-based, direct mutation testing [33]. A variety of methods are used to detect genetic mutations, including probe amplification refractory mutation systems [34,35], sequencing [36], high-resolution melting curve analysis techniques [37], and denaturing high-performance liquid chromatography [38]. Quantitative PCR has high sensitivity and specificity, but it is not conducive to clinical application due to the expensive equipment, strict quality control, and professional knowledge of molecular detection technology of PCR [28]. Among them, Sanger sequencing is the “gold standard” for mutation detection but is subject to sampling error and requires many resources outside of many diagnostic pathology laboratories [32]. IHC for BRAF (V600E), which tests for protein expression, is an excellent alternative, more sensitive and specific than Sanger sequencing in routine diagnostic settings, and may represent a new gold standard for the detection of BRAF (V600E) mutations in PTC [32]. Standard therapy with surgical resection and radioactive iodine ablation fails in about 10% of differentiated thyroid cancer. Preclinical and clinical studies for advanced thyroid cancer with BRAF (V600E) inhibitors, such as vemurafenib and dabrafenib, have demonstrated a significant clinical benefit [4].

Deep neural networks are powerful algorithms that can be applied to large images, such as H&E-stained whole slide images (WSIs) of tissues, with modest computing power. DL models can accurately diagnose cancer and identify cancer subtypes directly from histopathological and other medical images [23,39,40,41,42]. Anand et al. [43] presented a deep learning system using H&E-stained slides to predict BRAF mutation in thyroid cancer. Because most thyroid cancers are diagnosed using FNA, we try to use Artificial intelligence (AI)-based learning system in early determination of the BRAF status from cytological slides. In comparison with histopathological slides, Fine needle aspiration (FNA) has the advantages of speed, convenience, decreased cost, minimal morbidity, and a theoretically lower risk of local contamination [44,45,46,47]. However, downsides of FNA include inaccessibility of some masses, and variable accuracy, especially in diagnosis of sarcoma. In regard to FNA of general soft tissue masses, the literature reports a wide range of sensitivities (86–100%), specificities (36–100%), and diagnostic accuracies (21.9–98%) [45,46,47,48,49,50,51,52,53,54,55,56,57,58,59,60,61,62,63]. This study aims to build a reliable, accurate and cost-effective approach in early determination of the BRAF status from cytological slides. AI is rapidly reshaping cancer research and personalized medicine. DL-based models that accurately diagnose cancer and identify cancer subtypes directly from histopathological images [64]. Another area of interest for AI is the detection of certain key mutations directly from histopathology images, especially clinically actionable mutations (such as activating mutations in EGFR) as biomarkers of response to targeted therapies [64]. DeepPATH was able to identify six key mutations, including STK11, EGFR, FAT1, SETBP1, KRAS, and TP53 in lung cancer WSIs [42]. The compelling idea is to predict microsatellite instability (MSI) status directly from H&E-stained histopathology images, which are readily available; this would provide a more economical and time-saving alternative to existing methods, such as qPCR, IHC, or Next Generation Sequencing. Kather et al. [65] successfully applied a ResNet18 Convolutional Neural Network (CNN) in gastric, colorectal, and endometrial cancers to detect tumor regions in H&E slides and then classify them as MSI or microsatellite stable. Screed et al. [66] applying CNN (MobileNetV2 architecture) to the H&E-stained WSI of resected tumors can predict the prognosis of patients with chemotherapy and/or radiotherapy in early-stage colorectal cancer. We have previously proposed an automated DL framework for the identification of PTC from both FNAC and TP slides [67] and provided an automated weakly supervised DL framework for selection and guidance of bevacizumab-targeted therapy in epithelial ovarian cancer and peritoneal serous papillary carcinoma patients by analyzing H&E stains and immune-related biomarkers, such as AIM2, NLRP3, C3, and C5. The H&E stains and proposed-AIM2 model are demonstrated to be useful for bevacizumab therapeutic prediction [68,69,70]. So-called BRAF mutations are typical of PTC. The most important BRAF mutation BRAF (V600E), is found in approximately 45% of PTC. A meta-analysis showed that FNA-verified BRAF(V600E) mutations are associated with a risk of 99.3% for PTC [71]. Routinely used cytological images are a potential window into genomic signatures and may prove useful in predicting specific clinically meaningful molecular signatures without the need for tumor sequencing [72]. However, routine analysis of smears is currently not possible, not least because this approach would increase the costs. We apply the AI learning process in BRAF (V600E) prediction with high accuracy, precision, sensitivity, specificity and mean SS, rather than focusing on the traditional cytological evaluation of PTC detection. DL has the potential to dramatically affect nearly all aspects of tumor cytology-from enhancing diagnosis to personalizing treatment and discovering novel anticancer drugs. The proposed CLAM based approach is data efficient and also can generalize to multi-class classification and subtyping problems in addition to the binary tumour versus normal classification tasks typically studied in weakly supervised fashion [24]. This study presents a computational pathology framework that extends attention-based multiple-instance aggregation [73] to general weakly supervised WSI classification without requiring any pixel-level annotation, ROI extraction or sampling. The proposed cost-effective technology might be available in all Centers involved in the research of thyroid carcinoma in the future. In the future, we hope that the system could be applied in clinical and would ideally extend the system to other types of smear, like pleural effusion and ascite.

## Figures and Tables

**Table 1:** Quantitative evaluation for classification of BRAF (V600E) results in thyroid FNA and TP slides.

Methods	Accuracy	Precision	Sensitivity	Specificity	Mean SS (Sens., Spec.)
NASNetLarge [22]	0.82	0.82	1.00	0.00	0.50
MIL with Resnet34 + RNN [23]	0.77	0.83	0.90	0.14	0.52
CLAM with Resnet50 [24]	0.82	0.86	0.94	0.29	0.62
Modified CLAM with Resnet101	**0.87**	**0.94**	0.91	**0.71**	**0.81**

## Data Availability

The data that support the findings of this study are available from the corresponding author upon reasonable request.
